# Editorial: Autoimmune blistering diseases: advances in the understanding of pathogenesis and new therapeutic horizons

**DOI:** 10.3389/fmed.2023.1243878

**Published:** 2023-07-12

**Authors:** Giulia Gasparini, Kyle T. Amber, Emanuele Cozzani, Aurora Parodi

**Affiliations:** ^1^Section of Dermatology, Dipartimento di Scienze della Salute (DISSAL), University of Genoa, Genoa, Italy; ^2^Dermatology Unit, Ospedale Policlinico San Martino Istituto di Ricovero e Cura a Carattere Scientifico (IRCCS), Genoa, Italy; ^3^Department of Dermatology, Rush University Medical Center, Chicago, IL, United States; ^4^Department of Internal Medicine, Rush University Medical Center, Chicago, IL, United States

**Keywords:** bullous pemphigoid, pemphigoid diseases, pemphigus, autoimmune blistering dermatoses, autoimmune bullous dermatoses

Autoimmune blistering diseases (AIBDs) are a heterogeneous group of diseases, which are characterized by the loss of immunotolerance toward skin antigens and autoimmune responses that result in the development of bullae and erosions on the skin and mucous membranes. Moderate to severe forms of AIBDs often require long-term systemic immunosuppression. Currently, therapeutic options are limited and mostly consist of broad-spectrum highly immunosuppressive agents, associated with significant side effects. High level immunosuppression can expose to more severe forms of infectious diseases. Pira et al. well describe in their review how the risk of COVID-19 infection and its severe course manifested in AIBD patients treated with rituximab as compared to the general population. Rituximab being one of the more immunosuppressive drugs used for AIBDs (1).

As compared to other immune-mediated dermatological diseases, such as atopic dermatitis or psoriasis, AIBDs lack specific targeted therapies. In recent decades, few advances in the treatment of AIBDs have been made, aside from the approval of rituximab for moderate and severe pemphigus vulgaris.

Therefore, the greatest unmet need in the management of AIBDs is the development and approval of new *targeted* therapeutic agents. The ideal therapeutic agent should not generate broad-spectrum immunosuppression but rather immunomodulate a specific altered pathway while maintaining a favorable safety profile.

Thus, better understanding the pathogenesis of AIBDs represents the first essential step toward developing new drugs. Translational medicine represents the key to discovering new therapeutic approaches for AIBDs. In this scenario, we developed this Research Topic on autoimmune blistering diseases (AIBDs) with the goal of highlighting the cutting-edge research on the pathogenesis and the emerging therapeutic approaches.

Developing *in vitro* and animal models of AIBDs is a one the fundamental approaches for investigating pathogenic mechanisms and also for initial testing of new drugs. Radine et al. evaluated in electron microscopy the ultrastructural desmosomal morphology in the human skin organ culture (HSOC) model injected with either anti-desmoglein (DSG) 1/3 single-chain variable fragment (scFv, termed Px4-3). The authors demonstrated that the HSOC pemphigus model is an attractive tool to unravel novel therapeutic targets (Radine et al.).

Currently, several clinical trials are ongoing for the two most common AIBDs, namely pemphigus and bullous pemphigoid (BP). On the other hand, for rarer AIBDs, such as epidermolysis bullosa acquisita, mucous membrane pemphigoid or paraneoplastic pemphigus, specifically designed interventional clinical trials are scant. This phenomenon is reflected also in the research trends on AIBDs. Huang et al. analyzed with a bibliometric study the trending topics in the field of pemphigoid diseases and highlighted that most publications focus on molecular mechanisms and disease management, but 70% of the studies focus on BP while only a minority investigated other AIBDs belonging to the pemphigoid group.

New drugs targeting various pathways are currently being studied for the treatment of BP. The main targeted pathways are: type 2 immune response (both innate and adaptive; e.g., IL4/IL13, IL5, and eosinophils), immunoglobulins production and catabolism [B cells, circulating IgEs, and neonatal Fc receptor (FcRn)], complement cascade and IL-17 axis.

Zeng and Murrell thoroughly describe in their review article, how type II immunity, in particular eosinophils, are at the core of BP pathogenesis, and how other pathways, including IL-17/23 axis and complement, converge on this mechanism, making it a promising therapeutic target in BP. Nonetheless, not all studied targeted therapies for eosinophils gave satisfactory results. IL-5 is as a critical cytokine for eosinophilic maturation and functional activity, and it was demonstrated to play a relevant role in eosinophils function in BP (2–4). However, a randomized placebo-controlled, double-blind phase 2 pilot study on mepolizumab, an anti-IL-5 antibody (NCT01705795) was not effective in the treatment of BP (5).

In our Research Topic, Drenovska et al. report one of the few case series on the inhibition of the IL-17 in BP patients. The three patients were affected by both bullous pemphigoid and psoriasis, justifying the use of secukinumab. Notably, the authors show mixed results, with a positive clinical response of BP to secukinumab in two cases and the worsening of the blistering disease in another. The literature, as well, reports conflicting findings. Case reports and case series on successful biologic therapy of concomitant BP and psoriasis include one case treated with etanercept (6), one with ustekinumab (7), two cases with Ixekizumab (8, 9), and two cases with secukinumab in combination with prednisolone (10, 11). This must be weighed against reports of biologic therapies inducing the development of BP (12). However, a recent clinical trial of ixekizumab, another anti-IL-17A biologic agent approved for the treatment of psoriasis, failed to achieve the primary and secondary endpoints in the treatment of BP (NCT03099538) (13). Even more so, proper randomized trials are needed to establish the efficacy of IL-17 inhibition in BP.

Aside from BP, our Research Topic also included a therapeutic pearl for a very rare AIBD: papraneoplastic pemphigus (PNP). Chen et al. report a patient who was diagnosed with PNP and bronchiolitis obliterans (BO), the end stage life-threatening respiratory involvement of PNP, concurrent with chronic B lymphocytic leukemia (CLL). The patient was successfully treated with ibrutinib (Chen et al.). Ibrutinib is an irreversible inhibitor of Bruton's tyrosine kinase (BTK) that targets B cells and induces long-term remissions in B cell malignancies. By reducing B-cell proliferation and the production of proinflammatory cytokines, it has been shown to be an effective treatment option in patients with B-CLL -associated PNP (14). In contrast to a previous study (14), the Authors found a sustained response to ibrutinib with longer patient survival.

In conclusion, the development of new drugs necessitates research on molecular mechanisms, aided for example by disease models, real life experience on repurposing existing drugs on small case series, and clinical trials to finally validate the efficacy of new drugs. This process is not always linear nor successful. Sometimes, drugs that were promising on paper gave unsatisfactory or controversial results in clinical settings. Theoretical inhibition of pathogenic pathways does not necessarily translate into clinical efficacy. Our Research Topic collected remarkable contributions from all over the world, including Australia, China, Taiwan, Germany, Bulgaria, and Italy, strengthening the AIBDs research network. We also successfully met our goal of finding articles on the advances in the understanding of pathogenesis and new therapeutic horizons of AIBDs.

## Author contributions

GG and EC contributed to conception of the article. GG wrote the first draft of the manuscript. KA, EC, and AP revised and edited the final version. All authors contributed to the article and approved the submitted version.
